# Usage of Telehealth and Telenutrition Services by Registered Dietitian Nutritionists: Cross-Sectional Study

**DOI:** 10.2196/80211

**Published:** 2025-11-27

**Authors:** Najlaa Mohammed Al-Mana, Suhair Abdalla Abdalla, Asrar Abdulrahim Qari, Mohamed Eldigire Ahmed, Wejdan Saeed Alshehri, Lujain Salem Baabdullah

**Affiliations:** 1 Clinical Nutrition Department, College of Applied Medical Sciences, King Saud Bin Abdulaziz University for Health Sciences-Jeddah, P.O. Box 9515, Jeddah, Saudi Arabia, +966 122246346; 2 King Abdullah International Medical Research Centre, Ministry of the National Guard Health Affairs, Jeddah, Saudi Arabia; 3 Department of Basic Sciences, College of Science and Health Professions, King Saud Bin Abdulaziz University for Health Sciences-Jeddah, Jeddah, Saudi Arabia

**Keywords:** telehealth, dietetics, registered dietitian nutritionist, RDNs, nutrition education, telenutrition, COVID-19, Saudi Arabia

## Abstract

**Background:**

The COVID-19 pandemic has boosted telehealth adoption among clinical nutritionists globally. However, there is a research gap in Saudi Arabia concerning telehealth’s prevalence and effectiveness in dietetics practice.

**Objective:**

This study aims to evaluate telehealth implementation during the pandemic in Saudi Arabia.

**Methods:**

In this cross-sectional study, a web-based survey was used and distributed in several Saudi Arabian regions between December 2022 and May 2023. A convenience sample of 306 clinical registered dietitian nutritionists (RDNs) in public and private health care facilities who met the study’s inclusion criteria was included in this study.

**Results:**

During the COVID-19 pandemic, 56% (172/306) of RDNs used telehealth, showing significant differences in sociodemographics and telehealth knowledge at health care facilities (*P*=.04). Notable gender disparities were observed in years of experience, with 78% (61/78) of male dietitians working in public hospitals (*P*=.001 and *P*<.004). The main telehealth nutrition services provided included nutrition education (21%, 64/306), nutrition counseling (19%, 58/306), and nutrition monitoring (17%, 52/306). Telenutrition purposes primarily focused on nutrition education (21%), supporting weight and diet management (17.15%, 15%), and the management of chronic disease (14%, 43,306). Additionally, a smaller percentage of RDNs (8%-9%) used telehealth for the nutrition care process and health assessment, while no respondents reported using telehealth for sport nutrition services. Overall, 90% (275/306) of RDNs reported that they routinely incorporated telehealth into their practice. Common obstacles reported by RDNs using telehealth were internet connectivity issues (46%), difficulties in coordinating with patients (22%), and patient disengagement with a lack of motivation (13%).

**Conclusions:**

Our findings underscore the increasing adoption of telehealth by RDNs during the pandemic, highlighting its crucial role in nutrition services. The study suggests that technology enhancements and training initiatives can improve telehealth effectiveness, highlighting the need for further research in this dynamic field.

## Introduction

The integration of technology into health care has revolutionized how medical services are delivered and accessed. Telehealth, a key innovation in this domain, has gained significant traction over the past decade, driven by advances in digital technologies and the growing demand for accessible health care solutions [1]. Telehealth is broadly defined as the delivery of health care services and information through virtual and telecommunication technologies to facilitate long-distance clinical care [2]. Tools such as video conferencing, mobile applications, and electronic communication platforms exemplify the diverse applications of telehealth [3].

Numerous studies have highlighted the advantages of telehealth, including reduced hospitalization rates, shorter lengths of stay, and enhanced continuity of care [4]. Telehealth has also played a pivotal role in protecting patients and health care providers by minimizing physical contact during infectious disease outbreaks [5]. Beyond its safety benefits, telehealth addresses critical barriers in health care delivery by reducing financial burdens, overcoming geographical constraints, and fostering better usage of patient resources. Additionally, telehealth facilitates family involvement in care, even when family members are geographically distant [6].

Initially, telehealth adoption was limited primarily to primary care physicians. Over time, its application has expanded to include registered dietitian nutritionists (RDNs) and other health care professionals from various fields [7]. Dietitians can leverage telehealth for various purposes, including managing electronic health records, conducting consultations, and automating calculations such as BMI, calorie needs, and macronutrient distribution. Additionally, telehealth supports dietetic care beyond consultations by motivating patients, offering feedback, and enabling remote dietary monitoring [8]. The effectiveness of telehealth in dietetic care can be assessed based on various factors, including impact on dietitians’ workflows, patient satisfaction, utility, and time efficiency [9]. A survey conducted among members of British, Australian, and New Zealand dietetic associations revealed that 62% of dietitians used telehealth for nutrition-related applications, of whom 74% used them as information resources and 60% used them for patient self-monitoring [10]. Recent findings highlighted a notable increase in dietitians using social and mass media platforms for telenutrition, from 68.9% before the COVID-19 pandemic to 80% during it. Additionally, 20% more dietitians became active in sharing nutrition information online, with 40% spending over 4 hours daily on these platforms as part of their practice during the pandemic [11].

Evidence from randomized controlled trials has further underscored the potential of telehealth in nutritional counseling. For example, telehealth consultations have successfully enhanced nutritional awareness and improved dietary practices among patients with age-related macular degeneration [12]. The value of telehealth becomes particularly evident during infectious disease outbreaks. In the United States, the proportion of clinical nutritionists providing care through telehealth increased from 37% before the COVID-19 pandemic to 78% during it [13].

Research conducted in Lima, Peru, demonstrated that virtual consultations achieved outcomes comparable to in-person visits regarding anthropometric changes in overweight and obese adults. This finding supports the viability of telehealth as an alternative when necessary [14]. Telehealth has proven particularly valuable during infectious disease outbreaks by minimizing physical contact while maintaining the quality of care. In Saudi Arabia, studies involving neurologists and neurosurgeons reported high satisfaction with telehealth services during the COVID-19 pandemic. These health care professionals highlighted telehealth’s ability to foster stronger patient-provider relationships and enhance overall productivity. However, a notable challenge was the lack of physical examinations, which limited telehealth’s suitability for follow-up visits rather than initial consultations [15]. A study examining RDNs’ perspectives on telehealth found that 73.4% expressed optimism about its future role in dietetic practice [16]. However, further research is required to explore patients’ experiences with telehealth and identify obstacles and unmet needs [17].

In Saudi Arabia, the adoption of telehealth has mirrored global trends, with significant progress in recent years [18]. However, the vast geographical expanse of the Kingdom and the concentration of specialized health care services in urban centers present significant barriers to equitable access, particularly for nutrition services in rural and remote areas [19]. Before the pandemic, nutrition service coverage faced challenges, with studies indicating issues such as low dietary diversity intake [20], and varying levels of nutrition care services for conditions such as obesity [21]. These preexisting challenges underscore a substantial local need for accessible nutrition interventions. Despite these needs, there remains a notable gap in research examining the prevalence and effectiveness of telehealth in dietetic practice within the Kingdom. To bridge this critical gap and address the urgent need for accessible nutrition care, this study aimed to evaluate the application of telehealth in dietetic practice during the COVID-19 pandemic in Saudi Arabia. This research sought to provide valuable insights into how telehealth was adopted, the types of services delivered, and the challenges faced by dietitians, informing future strategies to optimize virtual nutrition care and enhance service accessibility across the country.

## Methods

### Study Design and Participants

To evaluate the use of telehealth in dietetics practice during the COVID-19 pandemic in Saudi Arabia, we conducted an anonymous, web-based cross-sectional survey between December 2022 and May 2023. The study was implemented across several regions of Saudi Arabia and targeted RDNs working in both public and private hospitals and clinics.

Convenience sampling was used. The survey was distributed primarily through professional groups of dietitians on WhatsApp and Telegram, as well as through hospital and clinic administration by sending the survey link to heads of clinical nutrition departments, who then disseminated it to eligible staff. Additionally, outreach was conducted via targeted emails to RDNs and relevant professional associations to maximize participant reach.

A total of 306 participants aged 24 to 59 years were included, consisting of 257 females (84.0%) and 49 males (16%). Inclusion criteria were RDNs with a minimum of 1 year of professional experience who had used telehealth (virtual) as well as in-person visits in their practice. Exclusion criteria were dietitians with less than 1 year of experience, those who had not used telehealth, academics, and students in clinical nutrition departments.

### Ethical Considerations

The study received ethical approval from the Ethics Research Committee at King Saud bin Abdulaziz University for Health Sciences (H-01-R-005). All participants were informed of the study’s purpose, provided a declaration of consent, and gave informed consent before completing the survey. Participation was entirely voluntary, and no compensation, incentives, or reimbursements were offered. All procedures were conducted in compliance with the ethical guidelines and regulations established by Saudi authorities.

### Survey Questionnaire

The survey was developed using Google Forms and was available in both Arabic and English. The questionnaire was adapted from a previously validated instrument [16] and modified to suit the local context. To ensure content validity, the survey was reviewed by clinical nutrition experts and refined based on feedback from a pilot test conducted with a group of dietitians similar in demographic profile to the target population. Items were revised for clarity and relevance as necessary. The reliability of the study scale was assessed by Cronbach ɑ coefficient for internal consistency, and the value was calculated at .74 showing a good level of internal consistency among scale items.

The questionnaire included sections on demographics (eg, age, gender, nationality, location, education, employment status, and patient population served), experiences with telehealth, perceived effectiveness, and barriers encountered during telehealth use.

### Data Collection, Storage, and Security

Data were collected via secure, password-protected Google Forms. The responses were stored in encrypted digital files accessible only to the research team. All data collection and storage procedures were designed to maintain participant confidentiality and were conducted in accordance with institutional and national data protection policies. Only aggregated, anonymized data were used for analysis, and no personally identifiable information was collected. All research activities adhered strictly to the applicable ethical and data security guidelines.

### Statistical Analysis

All collected data were cleaned and exported to IBM SPSS Statistics software (version 24.0) for analysis. A descriptive analysis was generated for all variables, with the quantitative variables being presented as mean and SD, and qualitative data reported in terms of frequency and percentage. Associations were tested using the *χ*^2^, Fisher exact test, and Kruskal-Wallis test depending on the nature of the variable. A *P* value of .05 or lower was considered significant for all analytical tests.

## Results

### Overview

A total of 481 participants responded to the telehealth in dietetic practice survey, and 175 participants were excluded due to missing data, resulting in a total sample size of 306 participating RDNs who reported they have worked in both public and private health care institutions, had been in practice for over a year, and had experiences in both virtual and in-person visits were included in the study.

### Sociodemographic Characteristics and Use of Telehealth at the Facility

Participant demographics, sociodemographic, and profession-related characteristics of dietitians working in hospitals and clinics in Saudi Arabia are presented in Table 1. Participant age ranged from 24 to 59 years, with 90% of them within the 24 to 34 years age range; the majority of them were female (257/306, 84%) with more than 84.3% of the RDNs holding a bachelor’s degree (n=258).

**Table 1.** Sociodemographic characteristics of RDNs and use of telehealth among study sample (N=306).

Sociodemographic characteristics	Sex, n (%)		Fisher exact test	Chi-square *(df)*	*P* value	Overall
	Female (n=257)	Male (n=49)				
Age (y), n (%)			4.94^a^	—^b^	.089	
24‐34	236 (91.8)	40 (81.6)				276 (90.2)
35‐44	16 (6.2)	7 (14.2)				23 (7.5)
45‐54	3 (1.2)	1 (2.0)				4 (1.3)
55‐59	2 (0.8)	1 (2.0)				3 (1.0)
Nationality, n (%)			1.55^a^	—	.30	
Non-Saudi	17 (6.6)	1 (2.0)				18 (5.9)
Saudi	240 (93.3)	48 (98)				288 (94.1)
Province, n (%)			^—^	32.95 (4)^c^	<.001	
Central Province	102 (39.6)	22 (45)				124 (40.5)
Eastern Province	46 (18)	4 (8.2)				50 (16.3)
North Province	7 (2.7)	3 (6.1)				10 (3.3)
South Province	8 (3.1)	11 (22.4)				19 (6.2)
Western Province	94 (36.5)	9 (18.3)				103 (33.7)
Highest level of education, n (%)			4.44^a^	—	.108	
Bachelor’s Degree	220 (85.6)	38 (77.6)				258 (84.3)
Doctorate	4 (1.6%)	3 (6.1)				7 (2.3)
Master’s degree	33 (12.8%)	8 (16.3)				41 (13.4)
Employment setting, n (%)			—	11.0 (2)^c^	.004	
Other	87 (33.8)	8 (16.3)				95 (31.0)
Private	36 (14)	3 (6.1)				39 (12.7)
Public	133 (51.7)	38 (77.6)				171 (55.9)
Years of experience, n (%)			25.47^a^	—	<.001	
<1	79 (30.7)	2 (4.1)				81 (26.5)
1‐5	132 (51.3)	30 (61.2)				162 (52.9)
5‐10	28 (10.8)	10 (20.4)				38 (12.4)
10‐15	11 (4.3)	1 (2.0)				12 (3.9)
15‐20	3 (1.2)	4 (8.2)				7 (2.3)
>20	4 (1.6)	2 (4.1)				6 (2.0)
Is telehealth implemented at your institution during the pandemic? n (%)			—	15.1 (2)^c^	<.001	
Don’t know	79 (30.7)	2 (4.1)				81 (26.5)
No	44 (17.1)	11 (22.4)				55 (18.0)
Yes	134 (52.1)	36 (73.4)				170 (55.6)

a Fisher exact tests were conducted for age, educational level, nationality, and years of experience.

b not applicable.

c Chi-square tests for association were conducted for province, employment setting, and telehealth implementation

The distribution of RDNs across different provinces was as follows: (124/306, 40.5%) in the central province, (103/306, 33.7%) in the western region, (50/306, 16.3%) in the eastern province, (19/306, 6.2%) in the southern province, and (10/306, 3.3%) in the northern province. The practice settings using telehealth comprise a mix of private hospitals, specialist clinics, research centers, and government hospitals caring for all age groups. Moreover, 56% of the respondents work as RDNs in public hospitals, whereas approximately 39% work at private hospitals and clinics. In comparison to other groups, RDNs stated that they work with adults the most frequently (40%), and approximately half of RDNs had 1 to 5 years of dietetics practice, in terms of experience.

There were significant variations in years of experience among dietitians, with notable differences observed between genders. Female dietitians tended to have less experience, with 30.7% (79/257) having less than 1 year of experience and 51.3% (132/207) having 1 to 5 years of experience. In contrast, male dietitians showed a different distribution, with 20.4% (10/49) having 5 to 10 years of experience and a higher percentage having over 20 years of experience at a senior level. This disparity in experience levels between male and female dietitians was statistically significant (*P*=.001).

The study highlighted gender disparities in the implementation of telehealth during the pandemic. A higher proportion of male dietitians (73%, 36/49) used telehealth compared to female dietitians (52%, 134/257). Additionally, male dietitians were more prevalent in public hospitals, with 78% (38/49) of male dietitians working in this setting (*P*<.01).

### Telehealth Practice and Experiences During the COVID-19 Pandemic

During the COVID-19 pandemic, only 55.6% (170/306) of the RDNs confirmed they used telehealth at the clinic or hospitals, 18.0% (55/306) did not use any telehealth, whereas 26.5% (81/306) did not know whether telehealth was used or not. *χ*^2^ tests revealed a significant difference across the sociodemographic domains and knowledge of the use of telehealth at the health facilities (*P*=.04; Table 1).

### Nutrition Care Services Through Telehealth

**Figure 1.**
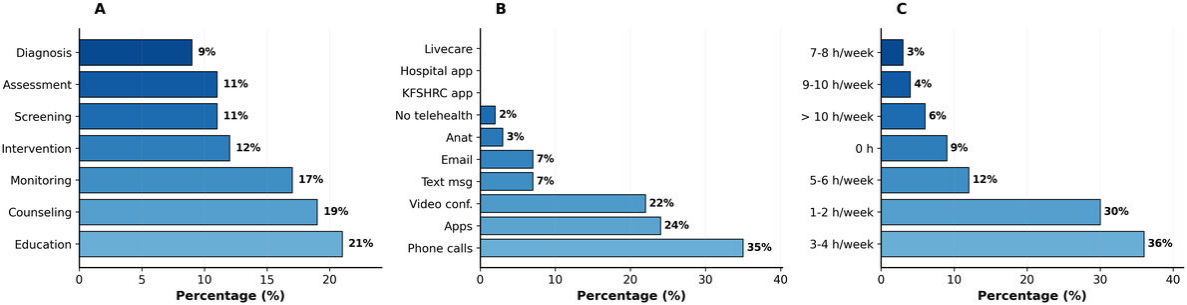
Telehealth services utilization during COVID-19. (A) Nutrition services offered, (B) types of applications used, and (C) frequency of use in clinical practice. Data shown as percentages (N=306).

According to Figure 1 (panel A), the majority of nutrition care services provided via telehealth during the COVID-19 pandemic included nutrition education, nutrition counseling, and nutrition monitoring. Respondents reported that typical aspects of the nutrition care process conducted through telehealth were nutrition education (21%, 64/306), nutrition counseling (19%, 58/306), and nutrition monitoring (17%, 52/306), with only 9% of study dietitians using telehealth for nutrition diagnosis.

Our results identified the most common telehealth platforms used by RDNs, including phones and video conferencing platforms such as Zoom and MS Teams. Among the respondents, 35%( 107/306) of RDNs reported providing nutrition intervention via telehealth through phone calls, followed by phone apps (eg, WhatsApp and Telegram) at 24% (73/306), and video conferencing (eg, Zoom and MS Teams) at 22% (67/306 ). Furthermore, only 7% (21/306) of RDNs indicated using emails or text messaging for nutrition care services, whereas a mere 3% (9/306) of RDNs used specialized applications such as Anat, a digital platform for health practitioners. Minimal usage (<1%) was observed for other institution-specific applications (Figure 1; panel B).

### Purpose and Frequency of Telehealth Implementation

When asked about the purposes of implementing telehealth, the study participants highlighted that the leading objectives were nutrition education 21% (64/306) and weight management 17.15% (53/306). Additionally, diet management at 15% (46/306) and the management of chronic disease at 14% (43/306) were noted as key purposes, with 8% to 9% (24-28/ 306) focusing on the nutrition care process and health assessment, and 5% (15/306) for administrative use. Interestingly, no respondents reported using telehealth for sports nutrition purposes. Furthermore, a significant proportion of RDNs mentioned that they typically used telehealth with their patients 90% (275/306). The survey indicated that more than half of the RDNs use telehealth services weekly, with a notable majority of 36% (110/306) of them dedicating an average of 3 to 4 hours per week to providing nutrition care services remotely (Figure 1; panel C).

### Obstacles and Challenges of Telehealth for RDNs

When using telehealth, RDNs encounter various obstacles and challenges across different employment settings. As per the findings presented in Table 2, the most prevalent obstacles experienced by RDNs during the implementation of telehealth were internet connection problems, affecting 46% (153/333) of participants. Interestingly, public settings reported higher rates of internet issues at 51% (78/170), with private settings close behind at 44%(17/39). Another significant challenge was coordination issues, with approximately 22% (73/333) of respondents facing difficulties in coordinating with patients during telehealth sessions. Public settings showed a slightly higher prevalence of coordination challenges at 25% (43/170), similar to other settings at 25% (24/95), whereas private settings experienced a lower rate at 4%(2/39). Approximately 13% (43/333) of RDNs reported patient disengagement and lack of motivation as a challenge. Notably, public settings had slightly higher instances of this issue at 11% (19/170) compared to private settings at 7% (3/39), whereas other employment settings reported 25% (24/95). Both patients and providers encountered technology-related challenges, with 6% (20/333) reporting patient technology issues and 6% (20/333) reporting provider technology issues. Non–internet-related technology issues were more pronounced in private settings, with 11% (4/39)and 4% (2/39) reported, compared to public settings at 5% (9/170) and 1% (2/170), and other settings at 8% (8/95) and 0%.

**Table 2.** Obstacles and challenges faced by registered dietitian nutritionists in using telehealth services.

Challenges faced when using telehealth services	Overall (n=333), n (%)	By employment setting, n (%)			*P* value^a^
		Public	Private	Other	
Internet connection issues	153 (46)	87 (51)	17 (44)	24 (25)	.20
Patient disengagement and lack of motivation	43 (13)	19 (11)	3 (7)	24 (25)	.20
Inability to coordinate with a patient	73 (22)	43 (25)	2 (4)	24 (25)	.20
Patient technology issues (non–internet related)	20 (6)	9 (5)	4 (11)	8 (8)	.20
Provider technology issues (non–internet related)	20 (6)	2 (1)	2 (4)	0 (0)	.20
Others^b^	40 (12)	12 (7)	12 (30)	16 (17)	.20

a Kruskal-Wallis rank sum test.

b Other: physical examination issues, patient focus challenges, missed calls and complaints, patient commitment, lack of time and focus, patient engagement variations, technical education needs, and information understanding.

The “Others” category, representing 12% (40/333) of challenges and obstacles encountered by RDNs, included a diverse range of issues such as physical examination limitations, patient focus difficulties, missed calls impacting scheduling, patient commitment affecting appointments, knowledge gaps in tube feeding, time and focus constraints, varied patient engagement levels, technical education needs, and challenges in understanding information accurately. These challenges were most frequently reported among respondents from private employment settings at 30% (12/39), compared to 7% (12/170) in public settings and 17% (16/95) in other settings.

## Discussion

### Principal Findings

The COVID-19 pandemic had a profound impact on health care delivery worldwide, accelerating the adoption of telehealth as a critical tool for patient care. This rapid shift toward telehealth prompted health care providers and patients to adapt to virtual consultations, remote monitoring, and other telehealth services. This study examines telehealth usage among RDNs, focusing on the mode and frequency of use, as well as challenges of online health care navigation via telehealth as a care modality. The broader context of telehealth adoption in emerging economies, as highlighted by studies, such as Assaad et al, provides a valuable framework for understanding the unique dynamics at play in the Saudi Arabian health care landscape [22].

Saudi Arabia’s health care system is characterized by a predominantly public-funded and public-owned structure, providing free universal health care to its citizens. While there is a growing private sector, the government remains the largest operator, with ongoing reforms under Vision 2030 aiming to enhance efficiency and introduce privatization initiatives [23]. This unique context significantly influences health care delivery and the adoption of new technologies such as telehealth. Telehealth practice among RDNs during the COVID-19 pandemic showed various patterns [23]. Telehealth practice among RDNs during the COVID-19 pandemic showed various patterns [24]. The study results revealed that more than half of the RDNs had used telehealth in clinics or hospitals, whereas 18% reported not using any telehealth services, and 26% were uncertain about whether telehealth was implemented at their health facilities. Interestingly, the variability in telehealth adoption among RDNs was significantly influenced by sociodemographic factors (*P*=.04).

Concerning the cross-sectional study on the use of telehealth in the COVID-19 pandemic, a gender disparity existed (*P*<.001). A greater proportion of male participants (73%) reported that telehealth was adopted by their institution, compared to female participants (52%). Additionally, 31% of females were unaware of telehealth implementation, compared to 4.1% of male participants. This knowledge gap indicates that male participants were more informed about the use of telehealth at their facilities. That said, because the vast majority of the sample was female participants, these results should be interpreted with caution. The gendered division of labor in Saudi health care is evolving, with increasing female participation, although disparities in leadership roles persist, influenced by societal norms [25,26]. This broader context of gender dynamics within the Saudi healthcare workforce may contribute to the observed differences in awareness and adoption of telehealth among male and female RDNs.

The 56% usage rate indicates that barely over half of the RDNs included telehealth into their practice during the pandemic, suggesting moderate acceptance and integrating telehealth technologies within the dietetics profession. This finding aligns with a recent study across 10 Arab countries by Bookari et al [11], which investigated dietitians’ perceptions and practices regarding social or mass media use during the transition to telenutrition. Furthermore, a regional cross-sectional study by Bookari et al [27] provided a snapshot of the experiences of dietitians during the COVID-19 crisis in 5 Arab countries, highlighting similar trends and challenges in telehealth adoption. While a similar level of telemedicine implementation was also observed among RDNs in Argentina during the COVID-19 pandemic [28], the Saudi Arabian context, with its public hospital dominance and ongoing health care reforms, presents unique challenges and opportunities for telehealth integration that warrant specific attention. In contrast, medical and mental health care providers rapidly adopted telemedicine to ensure that they continued to deliver care without violating social distancing protocols [29,30].

The 18% of RDNs who had never used telehealth suggest a subgroup within the profession that is inexperienced with or unwilling to integrate such technologies. This might be due to constraints in gaining access to technology, training deficiencies, or self-perceived inefficiency in providing nutrition counseling through technology. Expanding on these factors will be pertinent to fully identify the reasons for telehealth reluctance or inability [8]. These barriers may require more detailed training conducted through the telehealth platforms, the availability of relevant technological equipment, and proving the effectiveness of telehealth in the provision of dietetics.

In this study, we found that 26% of the RDNs were unable to accurately describe the extent of telehealth integration in their health care facilities. This highlights a significant issue in communication and information dissemination within the healthcare sector, suggesting a lack of awareness about telehealth services. To address this, it is crucial for decision-makers at different levels to enhance internal communication and ensure that all health care professionals are well-informed about telehealth options and protocols [31].

Telehealth use during the pandemic also varied significantly by age. The majority of RDNs who used telehealth were between the ages of 24 and 34 years, compared to RDNs between the ages of 35 and 59 years who reported minimal use of telehealth. This is consistent with previous research indicating that younger generations are more willing and likely to learn and adopt new technologies [32-34], together highlighting a generational divide in technology adoption.

The levels of telehealth knowledge as functions of age, years of experience, and perhaps geographic location indicate potential factors that may affect or mitigate telehealth implementation. Younger or less experienced RDNs may adapt more easily to telehealth technologies, whereas more seasoned professionals may face challenges in using these tools. Similarly, access to telehealth functioning may be more readily available for RDNs in urban areas compared with rural contexts [35]. To address these disparities, measures must be taken to address barriers and concerns specific to different demographic groups [36]. The results present substantial provincial variations, the workplace distribution, with marked gender disparities.

The range of services delivered through telehealth by RDNs during the COVID-19 pandemic highlights the versatility of telehealth in nutrition practice. The data indicate that nutrition education was the most common service provided through telehealth (21%), nutrition counseling (19%), and nutrition monitoring (17%). These findings are consistent with previous studies by Law et al [37] and Kelly et al [38], which have emphasized the efficacy of telehealth in providing essential nutrition services. Our findings regarding nutrition monitoring in telehealth parallel research by Gnagnarella et al [31], who found that RDNs can use telehealth to document patients’ progress and adherence to prescribed diets. Moreover, telehealth has enabled continuous nutrition monitoring, as supported by the work of Krznarić et al [39], who found that remote monitoring tools have significantly enhanced patient adherence to nutrition plans and improved health outcomes. Emphasizing the importance of these services in addressing patients’ dietary and health needs, particularly in challenging times such as the pandemic [40,41]. The results of this study, alongside the previous research, underscore the adaptability and potential of telehealth in delivering comprehensive nutrition care services, ensuring that patients receive uninterrupted and quality care during unprecedented times.

The study reflects a strategic use of telehealth platforms by RDNs to maintain patient engagement and deliver essential nutrition services during the pandemic. Communications for nutrition education and follow-up consultations were primarily conducted via phone calls (35%), followed by phone applications (34%) and video conferencing tools (22%). Despite difficulties such as connectivity problems or patients’ nonengagement, RDNs mostly described their work as enjoyable and promising, perceiving telehealth as future-oriented clinical work. While text and email communication were less common (7%), indicating a preference for more interactive channels, other platforms and institution-specific apps had minimal usage (<1%). These results align with studies in the United States and China indicating up to 53% use of technologies for communication and patients’ follow-up routines [42,43].

The focus on nutrition education and counseling services suggests that although telehealth was employed to uphold key interventions and serve patients’ continuing requirements, it remained most significant for ensuring that patients received the dietary guidance they needed while physical access was disallowed. This aligns with other research by Hammersley et al [44], who conducted a review on telehealth nutrition services, reporting that they mainly involved education and counseling to keep patients on track by following professional dietary advice. This is also supported by Birkhoff and Moriarty [45], who found that telehealth changed the patient experience in that education and support could always be conducted when face-to-face consultations were not feasible.

The data suggest that before the pandemic, telehealth was employed mainly for informative and counseling interventions, which are core components of nutrition management that may be implemented via distance. A review by Sanaeinasab et al [46] noted that nutrition education and counseling are the fundamental components of telehealth services. This supports the conception of the present study that these services are being prioritized to deliver dietary necessities to patients physically restricted from consultations. Together, these studies affirm the centrality of nutrition education and counseling in telehealth and support our conclusion that these services are essential to sustain patients’ care during disruptions such as the pandemic.

As expected, nutrition education was the most provided service, followed by weight management, diet management, and chronic disease management. These services are crucial in closely monitoring patients’ conditions from a distance. This emphasizes the necessity of accessible and convenient media, such as phone calls and applications, for patient interaction and further care [13]. Conversely, the delivery of more specialized services such as metabolic and sports nutrition was notably infrequent, constituting less than 1% of the services provided. This could be attributable to the complexity and specificity of these services, which often require detailed physical assessments and tailored interventions that are challenging to perform remotely. Therefore, although telehealth can be effective for general nutrition counseling, it may have limitations in addressing the nuanced needs of specialized nutrition fields. However, a recent study by Eid et al [47] demonstrates the potential of telenutrition weight loss programs supported by telemonitoring and telehealth coaching to achieve positive anthropometric and biochemical outcomes in overweight and obese adults. These findings underscore the importance of further developing telehealth capabilities to better accommodate a broader range of nutrition services, ensuring comprehensive care delivery in both general and specialized nutrition domains. Nevertheless, the inconsistencies of telenutrition services indicate that some domains require further enhancement and inclusion into overall telehealth systems for effective and adequate nutrition support [11].

The difficulties faced by RDNs varied across different employment settings, providing insights into their diverse needs and identifying key areas that require optimal support [5]. Several concerns were exhibited regarding the use of telehealth, which differed between public and private employees’ experiences. Internet connection issues were observed by 46% of RDNs, with the highest proportion in public (51%) as opposed to private (44%) and other (25%) practice settings, although not statistically significant (*P*=.2). Other drawbacks were physical examination, patients’ distractedness, missed appointments, and lack of patient-caretaker information, which were reported by 12% overall, with the “other” setting being the highest at 30%, public at 7%, and private at 17%. These findings underscore the importance of targeted approaches to address these issues that could enhance telehealth service experiences for various employment settings [15,48,49]. Ethics and privacy are critical in the field of telehealth. To address these issues that were not addressed in this study, the implementation of appropriate privacy and security measures that are designed to protect patient information during virtual consultation is a priority. Providing RDNs with training on ethical standards and privacy regulations related to telehealth will help to ensure the protection of patient privacy and confidentiality.

This study has notable strengths. To our knowledge, this study is the first to evaluate telenutrition use across various regions in Saudi Arabia. The survey’s validation and reliability were ensured by experienced RDNs practicing telenutrition, along with input from a focus group with diverse backgrounds, which helped ensure that the questionnaire was relevant and comprehensive for the target populations in special practice areas. However, this study has some limitations that should be considered when using the findings to develop telehealth practices and policies. First, the data collection was based on self-reports, which may be prone to respondents’ memory or reporting bias. Second, the study employed convenience sampling and distributed surveys primarily through social media and online channels. This approach, while efficient during the pandemic, may have introduced selection bias by favoring younger or more technologically adept participants, potentially underrepresenting older RDNs or those less comfortable with digital platforms. Third, the participation was voluntary, and population bias may thus be an issue. The cross-sectional nature of the research limits our ability to indicate any causality or changes over time. Additionally, some subgroup analyses were constrained by small sample sizes, limiting the generalizability of these results. Finally, the study did not explore relative issues related to particular barriers and facilitators of telehealth, reducing the possibility of understanding the difficulties and prospects of RDNs in various settings.

### Future Prospects

The future of telehealth in dietetics practice holds promising opportunities for innovation and advancement. To enhance the integration of telehealth, future efforts may focus on tailored training development, acquisition of technologies, and the effective internal communication in health care organizations. By incorporating emerging technologies, such as artificial intelligence and wearable devices, we may be able to elevate the delivery of personalized nutrition care and monitoring. The use of telehealth platforms in virtual nutritional assessments and meal planning that are tailored to the needs of the individual can revolutionize dietetic practice. There is a growing demand for further research to investigate the long-term effectiveness and outcomes of telehealth interventions in nutrition care. Future studies could focus on optimizing telehealth workflows, developing telehealth-specific training programs for RDNs, and exploring telehealth services to reach underserved populations. By embracing these future directions and implementing technological advancements, telehealth has the potential to reshape and elevate the quality standards of care in dietetics practice.

### Conclusions

Our study findings provide valuable insights that significantly contribute to the broader field of digital health in nutrition. The high usage of telehealth by RDNs during the COVID-19 pandemic underscores the growing importance and acceptance of digital health technologies in delivering nutrition care. While many RDNs used telehealth services effectively for education and counseling, several challenges were identified, including internet connectivity problems (affecting 46% of the respondents) and patients’ nonattendance.

To address these obstacles, it is recommended that health care institutions focus on strengthening digital infrastructure, particularly in areas with unreliable internet connections to ensure consistent and high quality of telehealth delivery. Additionally, developing standardized and clear protocols for managing virtual appointments, including effective patient reminders and follow-up systems to reduce no-show rates. The implementation of targeted training programs will not only enhance the technical skills of RDNs in using telehealth platforms but also equip them with strategies for troubleshooting network issues and maintaining patient engagement in virtual settings. The findings suggest a need for increased research and special interventions on evaluating the effectiveness of these interventions and on exploring innovative solutions to technological barriers in digital health. Addressing limitations and challenges will be a key to embracing telehealth for enhanced access to high-quality nutrition services for all individuals. Overall, these findings expand our understanding of the evolving landscape of digital health in nutrition and provide a foundation for further research and development in this rapidly advancing field.

## Abbreviations

RDN: registered dietitian nutritionist
